# Evaluation of gut microbiota alterations following orlistat administration in obese mice

**DOI:** 10.3389/fendo.2024.1337245

**Published:** 2025-02-26

**Authors:** Chang Xue, Tianying Wang, Yang Chen, He Zhang, Hongjie Wang, Qiang Li

**Affiliations:** ^1^ Department of Endocrinology & Metabolism, The Fourth Affiliated Hospital of Harbin Medical University, Harbin, China; ^2^ Clinical Research Center, Qingdao Municipal Hospital, Qingdao, China; ^3^ Department of Microbiology, Harbin Medical University, Harbin, China; ^4^ Department of Endocrinology & Metabolism, Shenzhen University General Hospital, Shenzhen, China

**Keywords:** orlistat, obesity, intestinal hormones, gut microbiota, high-throughput sequencing

## Abstract

**Background:**

The gut microbiota plays a pivotal role in various metabolic disorders. Orlistat has shown beneficial effects on weight loss and metabolism, but its direct impact on the gut microbiota has not been extensively reported. Thus, this study aimed to explore the effects of orlistat on the gut microbiota in mice with high-fat diet-induced obesity.

**Methods:**

Thirty male C57BL/6J mice were randomly divided into a normal control group (fed a standard diet, N), and a model group (fed a 60% fat diet). A body weight exceeding the basal body weight by 130% defined a successfully established obesity model. The model group was further divided into a positive control group (fed a 60% fat diet, F), and an orlistat group (fed a 60% fat diet and treated with orlistat at 30 mg/kg, bid, A), with 10 mice in each group. The parameters assessed included weight loss, fasting plasma glucose (FPG) levels, and intestinal hormones. Gut microbiota diversity was analyzed using high-throughput sequencing.

**Results:**

Orlistat treatment significantly reduced body weight and FPG levels, and increased glucagon-like peptide-1 (GLP-1) and gastric inhibitory polypeptide (GIP) levels in obese mice. High-fat diet-fed mice exhibited increased microbial diversity and richness, which were significantly diminished by orlistat administration. Additionally, orlistat treatment led to a significant decrease in the proportion of *Bacteroidetes* and an increase in the proportion of *Helicobacter* and *Allobaculum*. Notable shifts in the abundances of *Bacteroidetes* were observed, correlating with changes in several functional metabolic pathways, including “cell motility” and “neurodegenerative diseases.” Co-occurrence network analysis suggested a more complex bacterial network in orlistat-treated mice, alongside a reduction in the density of bacterial correlation networks.

**Conclusions:**

Our study demonstrates that orlistat’s beneficial effects on body weight, FPG, GLP-1, and GIP are likely mediated through modifications in the gut microbiota composition.

## Introduction

Obesity is a metabolic syndrome influenced by various factors, including diet and environment. Since 1980, the incidence of obesity has been escalating rapidly; nearly 2 billion adults are overweight, with more than half qualifying as obese (1). World Health Organization statistics indicate that overweight and obesity are linked to over 280,000 deaths annually (2). The “Report on the Nutrition and Chronic Disease Status of Chinese Residents” reveals that over 50% of Chinese adults are overweight or obese. Projected trends suggest that by 2050, the prevalence could rise to 40% among adult females, 60% among adult males, and 25% among children (3). Obesity is a principal contributor to other metabolic disorders, such as type 2 diabetes, cardiovascular diseases, and non-alcoholic fatty liver disease, all associated with increased mortality risk (4–6). Consequently, it is imperative to devise effective strategies to manage overweight and obesity to prevent chronic non-communicable diseases.

The gut microbiota is involved in obesity development, as it significantly affects the metabolic health of the human host. Gut microbiota dysbiosis can lead to various metabolic disorders, including type 2 diabetes, lipid abnormalities, obesity, and non-alcoholic fatty liver disease (7). In mammals, most microorganisms are integral to a complex microbial ecosystem essential for host immunity, metabolism, and productivity (8, 9). Obesity-related microbial dysbiosis is characterized by a decline in overall microbial diversity, an upsurge in sulfate-reducing bacteria and pathogens, and a reduction in beneficial SCFA-producing bacteria that promote health (10). The metabolic activities of the gut microbiota, such as influencing fat deposition, intestinal permeability, and chronic low-grade inflammation, may significantly contribute to obesity-related pathogenesis (11). Previous research has indicated a change in the ecological makeup of the gut microbiota in obese individuals, with an increased *Firmicutes*/*Bacteroidetes* ratio (12, 13). *Akkermansia muciniphila* has been identified as a pivotal component of the gut microbiota, playing a vital role in metabolic disorders, including obesity (14). Extensive research studies have been performed to identify interventions that can modulate the gut microbiota and yield positive effects on obesity (15–18).

The link between gut microbiota and obesity as well as other metabolic syndromes is becoming increasingly clear. Natural products are valued for their beneficial health effects in humans. An increasing number of studies have shown that the anti-obesity bioactivities of many natural products are dependent on the gut microbiota (19–22). Orlistat, a reversible inhibitor of gastric and pancreatic lipases, is extensively employed in the clinical management of obesity and its related complications. Long-term usage of Orlistat for over 12 months has been associated with an average weight reduction of approximately 2.9% (23). The hypothesized mechanism of Orlistat’s action includes impeding triglyceride hydrolysis, which leads to a decrease in fat absorption by about 30% (24). Given Orlistat’s commendable safety profile, it has gained approval for prolonged use in the weight management of individuals with obesity (25, 26). However, the effects of Orlistat on gut hormones and microbiota require further investigation. The primary hypothesis of this study is that Orlistat administration will modulate the gut microbiota composition in obese mice, leading to beneficial effects on body weight, fasting plasma glucose (FPG) levels, and intestinal hormones such as glucagon-like peptide-1 (GLP-1) and gastric inhibitory polypeptide (GIP). To test this hypothesis, we evaluated the changes in gut microbiota diversity and composition, as well as their correlation with metabolic parameters, in mice with high-fat diet-induced obesity.

## Methods

### Animals and treatments

Eight-week-old male C57BL/6J mice, weighing 18–20 g and classified as specific-pathogen-free (SPF), were housed in an SPF animal facility. The facility maintained a controlled environment with a room temperature of 22–24°C, 60% humidity, and a 12-hour light/dark cycle. Mice were health-screened before the start of the experiment to ensure they were free from any significant diseases or abnormalities. All mice were provided by The Animal Center at the Second Hospital of Harbin Medical University (Harbin, China). The experimental procedures adhered to the guidelines set forth by the Harbin Medical University’s Guide for the Care and Use of Laboratory Animals, and all protocols were approved by the Institutional Animal Care and Use Committee (IACUC). For one week, the mice were acclimatized and fed a complete diet comprising 40–43% corn, 26% bran, 29% bean cake, 1% salt, 1% bone meal, 1% lysine, 1% vitamins, and trace elements. During this period, their basal body weights were recorded to establish a baseline. After acclimatization, the mice were randomly divided into three groups: a normal control group (N, n=10) and a model group (n=20). The normal control group continued to receive the standard diet, while the model group was switched to a high-fat diet. The high-fat diet consisted of 60% fat and was composed of 2 kg of a mixture that included 1.2 kg of lard, 100 g of milk powder, and 0.8 kg of maltose, along with other components to ensure a balanced but high-fat intake. Following the establishment of the model, the 20 model mice were further randomly allocated into two subgroups: a positive control group (F, n=10) and an orlistat treatment group (A, n=10). The positive control group (F) received the high-fat diet without any additional treatments, while the orlistat group (A) received the high-fat diet supplemented with orlistat, a known anti-obesity drug, to evaluate its effects on weight gain and metabolic parameters.

Initially, the A received a 60% fat diet along with orlistat (30 mg/kg, twice daily, bid). The orlistat, provided by Hangzhou Zhongmei Huadong Pharmaceutical Co. Ltd. (China), was thoroughly pulverized and mixed with 0.5% carboxymethylcellulose (CMC) to ensure a uniform suspension. The mixture was subjected to repeated pipetting and ultrasonication to achieve a homogenous orlistat suspension, ensuring consistent dosing throughout the treatment period. The F group received a 60% fat diet and an equivalent volume of 0.5% CMC, which served as a vehicle control. This ensured that any observed effects in the orlistat group could be attributed to the drug itself rather than the vehicle. The N received a standard basic feed and an equivalent volume of distilled water, maintaining consistency in the experimental design. The treatment period spanned 9 weeks, during which all groups had unrestricted access to water and food.

### Body weight, fasting plasma glucose, intestinal hormones

Body weights were recorded weekly at the same time to ensure consistency and minimize variability. Initially, all mice underwent a 16-hour fast to standardize their metabolic state, followed by a 9-week feeding regimen according to their respective dietary groups. Capillary blood samples were collected from the distal third of the mice’s tails at baseline and after 9 weeks. Blood collection was performed under rapid ether anesthesia to minimize stress and discomfort. FPG levels were measured using a blood glucose meter (Jiangsu Yuyue Medical Equipment & Supply Co. Ltd., China).

At the conclusion of the experiment, anesthesia was induced with 3% isoflurane mixed with 30% oxygen and 70% nitrous oxide using an anesthetic chamber. Anesthesia was maintained with 1.5% isoflurane via a facemask to ensure that the mice remained fully anesthetized throughout the procedure. Under full anesthesia, cardiac blood collection was performed to obtain a sufficient volume of blood for further analysis. The mice were then promptly sacrificed by cervical dislocation to ensure a quick and painless death, minimizing any potential distress and ensuring the accuracy of the experimental results. Heart blood samples were collected, and serum was isolated by allowing the blood to clot at room temperature for 4 hours. The clotted blood was then incubated at 4°C for 12 hours and subsequently centrifuged at 3,000 rpm for 15 minutes to separate the serum. The isolated serum was stored at -80°C until further analysis to maintain the integrity of the samples. Serum GLP-1 and GIP levels were determined using an enzyme-linked immunosorbent assay (ELISA) kit (Bioswamp, Wuhan, China).

### DNA extraction of fecal bacteria

From the onset of the study (week 0) to its conclusion (week 9), mice from the three groups were placed into respective metabolic cages at predetermined intervals each week. Fresh feces from 10 mice across the groups were collected within 2 hours using sterile forceps, then stored in aseptic centrifuge tubes. The tubes were sealed and labeled with sealing film, and the samples were immediately frozen at -80°C to preserve the integrity of the microbial DNA. Fecal samples collected at weeks 0, 3, 6, and 9 were used for DNA analysis. DNA was extracted from the fecal samples using the QIAamp^®^ Fast DNA Stool Mini Kit (QIAGEN Biotechnology) according to the manufacturer’s instructions. The purity of the extracted DNA was assessed by measuring the absorbance of 12 randomly selected DNA samples using a Thermo NANODROP 2000C DNA detector. The DNA samples were then stored at -20°C in labeled centrifuge tubes to prevent degradation. The Qubit 2.0 DNA test kit was employed to quantify the isolated genomic DNA, which was subsequently used for PCR amplification. The PCR utilized primers fused with V3-V4 universal primers compatible with the MiSeq sequencing platform. The specific primers used were: i) 341F primer: CCCTACACGACGCTCTTCCGATCTG CCTACGGGNGGCWGCAG; 805R primer: GACTGGAGTTCCTTGGCACCCGAGAATTCCAGACTACHVGGGTATCTAATCC. PCR products from bacterial and archaeal DNA, with amplicon sizes over 400 bp, were treated with 0.6x volume of Agencourt AMPure XP magnetic beads to purify and size-select the DNA fragments. For fungal PCR products and other PCR products with amplicon sizes under 400 bp, 0.8x the volume of magnetic beads was used. This step ensures the removal of primer dimers and other non-specific PCR products, resulting in high-quality DNA for sequencing. The Qubit 2.0 DNA detection kit was used to accurately quantify the recovered DNA. The purified PCR products were then equally mixed at a 1:1 ratio to achieve a total of 10 ng of DNA per sample. This pooled DNA was prepared to a final sequencing concentration of 20 pmol, ensuring optimal conditions for subsequent sequencing on the MiSeq platform.

### High-throughput sequencing analysis

First, sample sequences were differentiated using barcodes. Each sample sequence then underwent a rigorous quality control process to remove non-specific amplifications and chimeric sequences (27, 28). Next, the raw reads were demultiplexed and allocated to their respective samples based on the barcode information. Vsearch (Version 2.10.4) was then utilized to merge paired-end reads from the initial DNA fragments. The merging process helps to reconstruct the full-length sequences from the shorter reads, which is essential for accurate downstream analysis. The merged raw tags were further refined into clean tags through the Vsearch quality control process. This refinement includes filtering out low-quality reads, trimming adapter sequences, and removing any remaining artifacts. The resulting clean tags are of high quality and suitable for subsequent analyses. To eliminate chimeric sequences, USEARCH (version 5.2.236) was employed using the *de novo* method. This approach identifies and removes chimeras that may have been formed during the PCR amplification process. Additionally, the Silva database was used to further screen and remove any remaining chimeric sequences (29).

Operational taxonomic units (OTUs) were analyzed by clustering sequences based on sequence similarity. Sequences were grouped into OTUs using a ≥97% similarity threshold, which is a common criterion for defining identical OTUs (30). A Venn diagram was used to display the number of shared and unique OTUs across the three groups, providing a clear illustration of their similarities and overlaps. This visual representation helps in understanding the distribution and uniqueness of OTUs among the different sample groups. The diversity of OTUs and their similarity indices were also evaluated to provide a quantitative measure of the microbial community structure. Additionally, a sample clustering tree diagram (dendrogram) was generated to provide a visual representation of the similarity and disparity among the samples. The dendrogram, depicted through its branching structure, shows how closely related the samples are to one another.

The alpha (within samples) and beta (among samples) diversities were analyzed using in-house Perl scripts, and the ggplot2 package in R (Version 3.2) was used for visualization. For each OTU, representative sequences were selected, and the GreenGenes database (Release gg_13_5) was used for taxonomic annotation. Dominant species classification based on abundance in each sample was performed using GraPhlAn 0.9.7 and iTOL 3.2.1 (31, 32). The predicted functional genes were categorized into Kyoto Encyclopedia of Genes and Genomes (KEGG) pathways and Kyoto Orthology (KO) groups. The differences in these functional profiles among the three groups were compared using STAMP (33). To determine the association between enriched taxa and significant functional metagenomes, Spearman’s correlation coefficients were calculated.

Principal Component Analysis (PCA) was employed to reduce the dimensionality of the dataset while preserving the most influential features that account for the variation. This method helps to discern key elements and structures, streamlining the complexity and elucidating underlying patterns. The PCA was implemented using the vegan package in R (Version 3.2). Additionally, a co-occurrence network analysis was conducted to explore the relationships and interactions between different microbial taxa. The nine microbiota OTUs with the highest relative abundances were selected and consolidated based on the lowest common taxonomy assignments at the genus level. Spearman correlation analysis was performed using non-rarified sequence data to identify significant associations between bacterial genera. An edge (connection) was created between two bacterial genera if the Spearman correlation coefficient met the criteria of P < 0.05 and |r| > 0.7. These thresholds ensure that only strong and statistically significant correlations are included in the network. The network analysis was conducted using R software (Version 3.2), which provides robust tools for visualizing and interpreting the complex relationships within the microbial community.

To trace the biological evolutionary sequence and comprehend potential mechanisms, a phylogenetic tree was constructed to analyze sequence differences at the taxonomic level. The multiple sequence alignment was performed using MUSCLE (Version 3.8.31) (34), which is a widely used tool for aligning multiple sequences with high accuracy and speed. Boxplots were generated to compare the distance distributions within and among groups. This visualization, created using R (Version 3.2), helps to illustrate the variability and central tendency of the distances, making it easier to identify any significant differences in the distribution of sequence distances between and within the sample groups. Lastly, the Linear Discriminant Analysis Effect Size (LEfSe) method was employed to identify differences in the relative abundance of taxa across the groups (35). LEfSe is a powerful tool that combines statistical significance with biological relevance by using linear discriminant analysis (LDA) to detect features (taxa) that are significantly different between groups.

### Statistical analysis

Body weight and metabolic indices are presented as mean ± standard deviation. One-way ANOVA was applied to compare the levels of body weight, FPG, intestinal hormones among different groups, *Post-hoc* pairwise comparisons were conducted using the Bonferroni correction method to control for the family-wise error rate and to identify specific differences between groups. All P values reported are two-tailed, with a significance threshold established at 0.05. Quantitative statistical analyses were executed using R software (Version 3.2).

## Results

### Body weight, fasting plasma glucose, intestinal hormones

**Figure 1:**
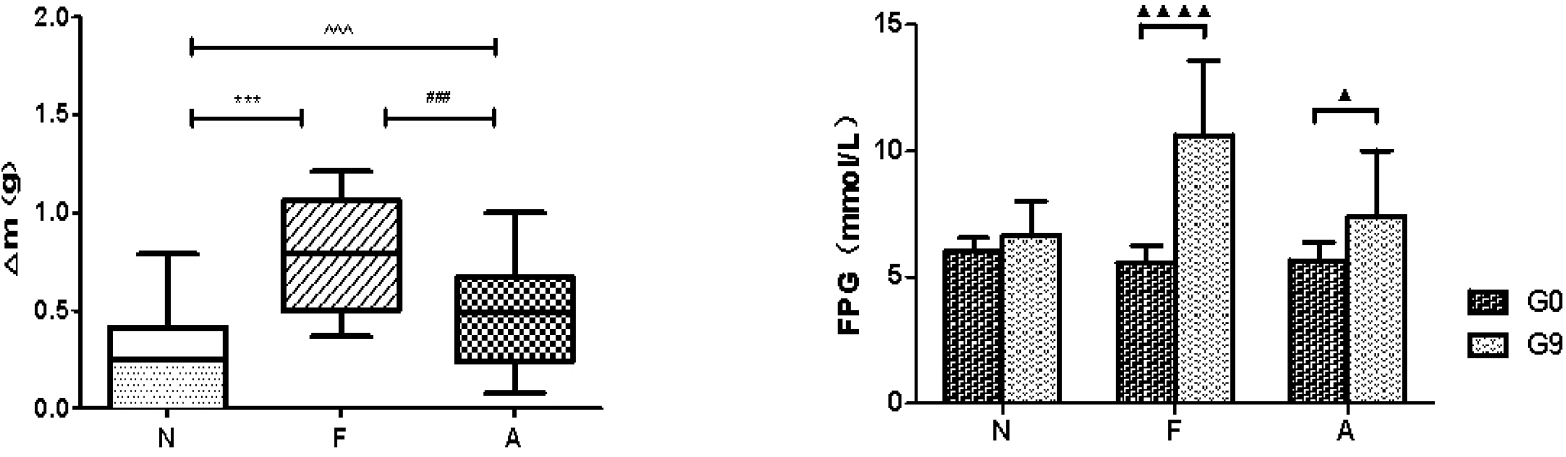
Average weekly weight change in three mice groups (left). N: Normal Control; F: Nutritional Obesity-Treatment Group; A: Nutritional Obesity Drug Treatment Group. F vs. N ^***^p<0.0001; A vs. F ^###^p<0.0001; A vs. N ^^^^^p<0.0001. Fasting plasma glucose (FPG) level comparison between week 0 and week 9 within the same group (right) N: Normal Control; F: Nutritional Obesity-Treatment Group; A: Nutritional Obesity Drug Treatment Group. G0: the first week, G9: the ninth week. ^▲^p<0.05, ^▲▲▲▲^p<0.0001. One-way ANOVA was used to evaluate the variations in body weight across different groups, followed by *post-hoc* pairwise comparisons using the Bonferroni method.

**Figure 2:**
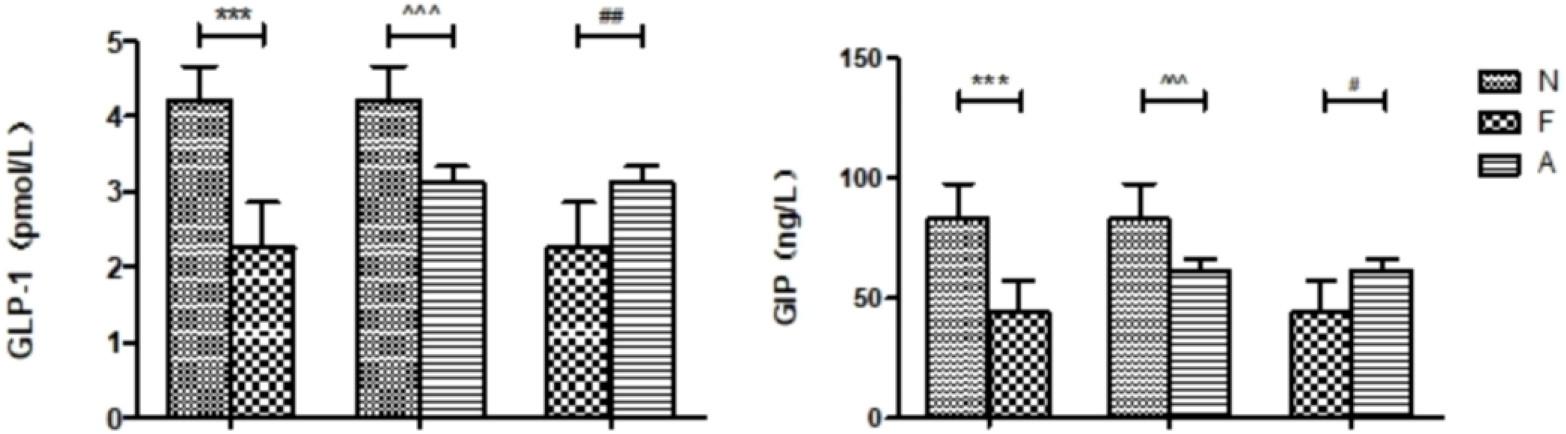
Comparison of various intestinal hormones between the three groups in the ninth week. N: Normal Control; F: Nutritional Obesity-Treatment Group; A: Nutritional Obesity Drug Treatment Group. F vs. N ^***^p<0.0001; A vs. F ^#^p<0.01, ^##^p<0.001; A vs. N ^^^^^p<0.0001. One-way ANOVA was applied to compare the levels of intestinal hormones among different groups, followed by *post-hoc* pairwise comparisons using the Bonferroni method.

The average weekly weight gain in the A group (0.48 ± 0.13 g vs 0.21 ± 0.12 g, P<0.0001) and the F group (0.78 ± 0.14 g vs 0.21 ± 0.12 g, P<0.0001) was significantly higher compared to the N group. In addition, the weight gain in the A group was significantly lower than that in the F group (0.48 ± 0.13 g vs 0.78 ± 0.14 g, P<0.0001; **Figure 1**). A significant elevation in FPG levels was observed in both F and A groups, with the A group showing a comparatively lower increase in FPG than the F group (**Figure 1**). Moreover, the levels of GLP-1 and GIP in the F and A groups were significantly reduced compared to the N group. Notably, the A group showed increased levels of GLP-1 and GIP relative to the F group, independent of weight changes (**Figure 2**).

### High-throughput sequencing analysis for gut microbiota

**Figure 3:**
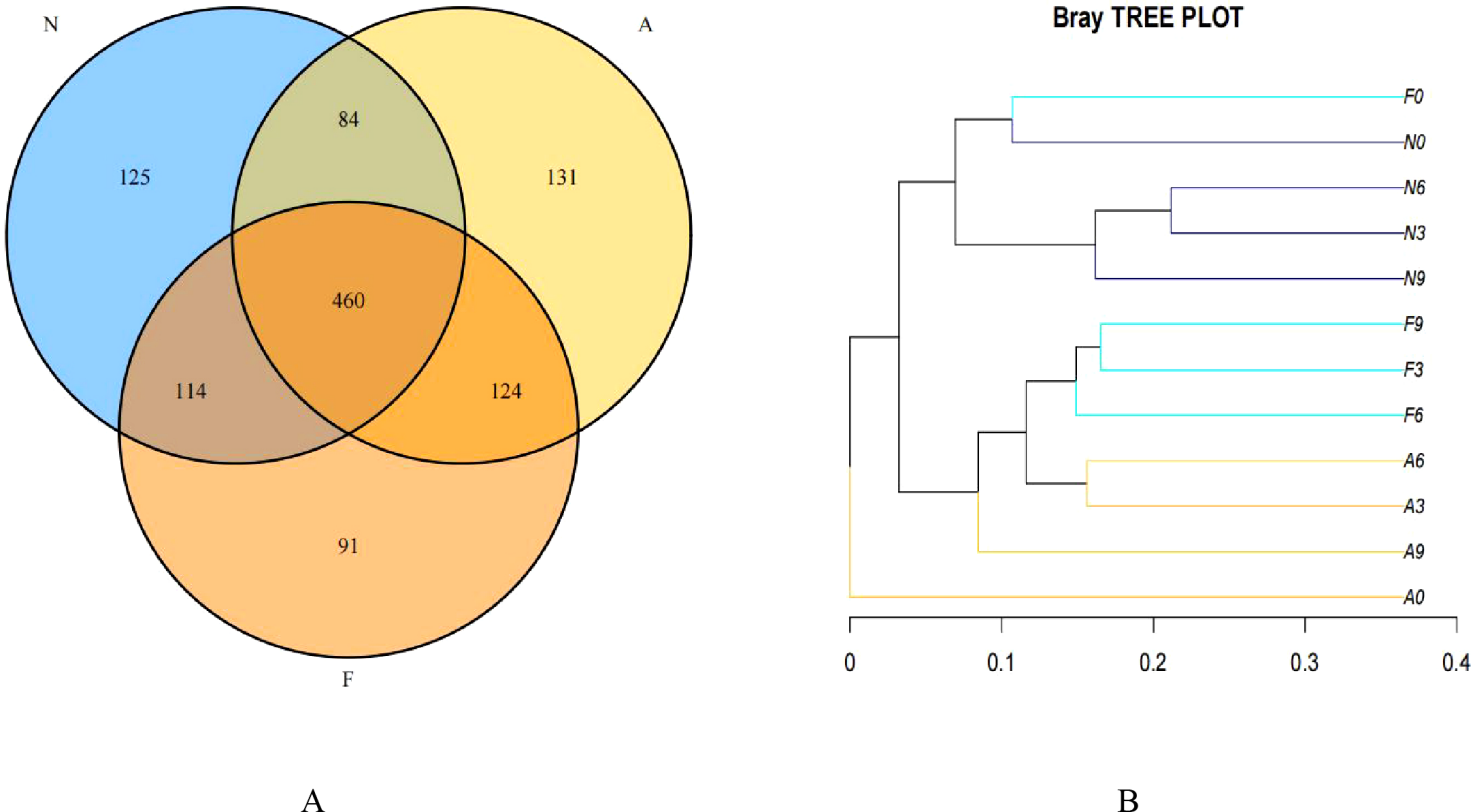
Venn diagram **(A)** and clustering tree **(B)** showing the similarity and overlap of operational taxonomic units (OTUs). The Venn diagram and clustering tree were generated using R software (Version 3.2).

**Figure 4:**
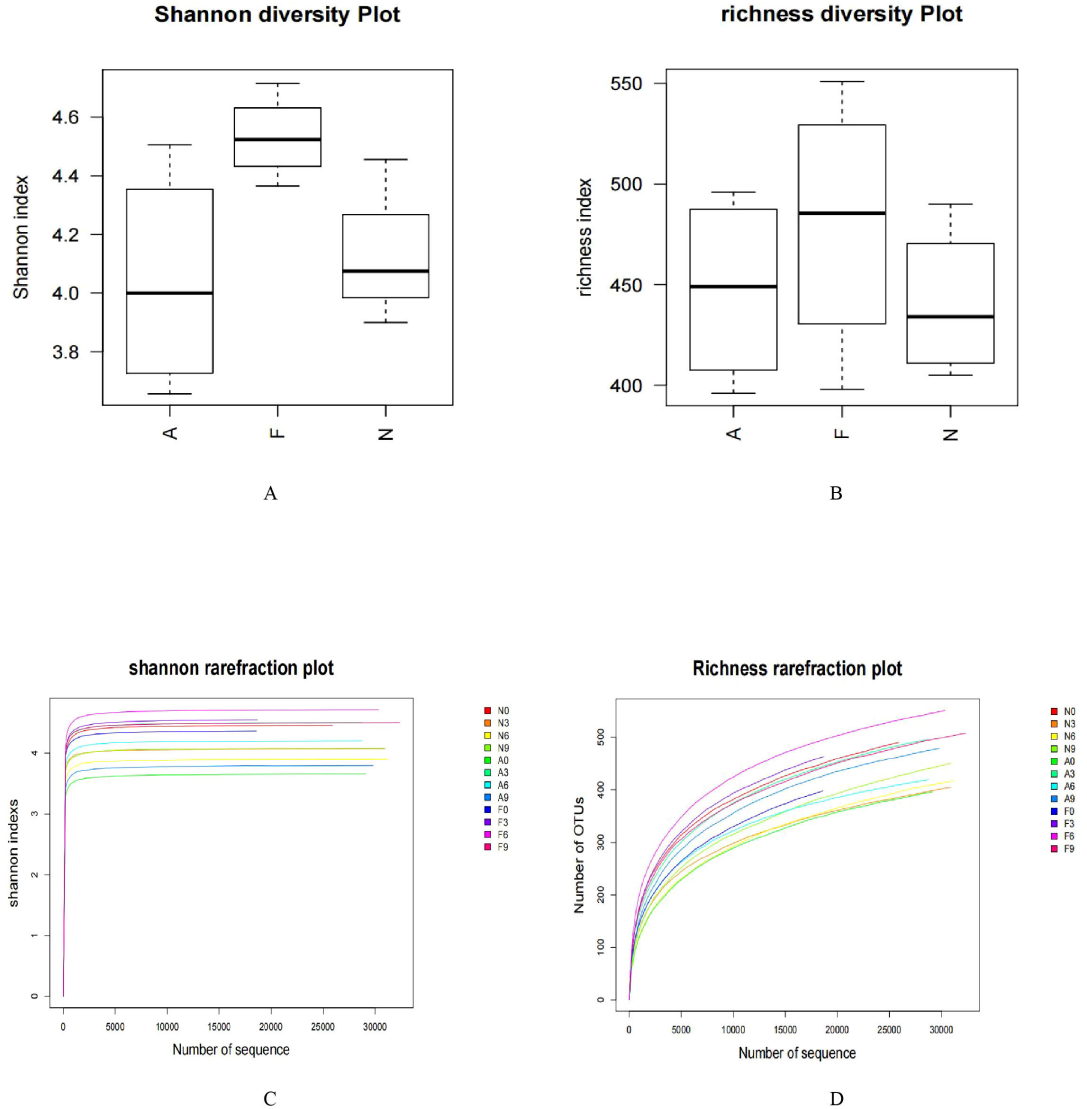
Impact of orlistat on the gut microbial composition in obese mice. Pairwise comparisons of α-diversity, including **(A)** Shannon index and **(B)** Richness index, across the N, F, and A groups. **(C)** Rarefaction curves for species diversity in the three groups. **(D)** Rarefaction curves for species richness (observed OTUs) in the three groups. The plateau of the curves indicates sufficient sequence sampling. One-way ANOVA was applied to compare the α-diversity indices among different groups, followed by *post-hoc* pairwise comparisons using the Bonferroni method.

**Figure 5:**
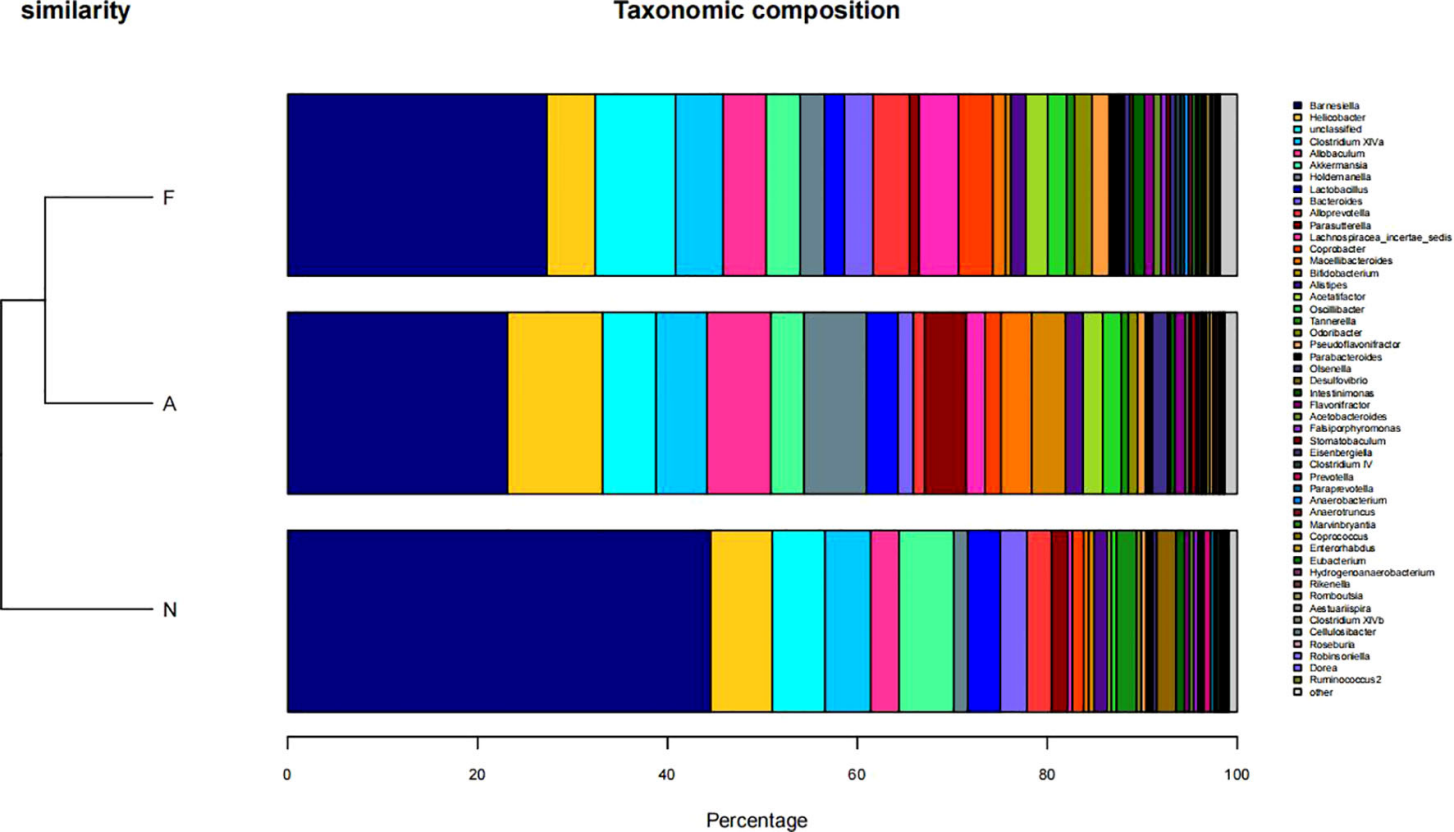
Taxonomic composition of microbial communities in fecal samples across three groups. The taxonomic composition was determined using R software (Version 3.2).

From 12 fecal samples (four from each group), a total of 360,612 valid sequences were procured. After data trimming and quality control, 343,894 high-quality sequences were retained, averaging 28,658 sequences per sample. **Figure 3A** displays the similarity and overlap in the number of OTUs across the three groups, while the clustering tree diagram shown in **Figure 3B** demonstrates a high similarity between the F0 and N0 groups. The microbial diversity and richness were higher in the F group compared to the N group. Nevertheless, orlistat treatment led to a decline in both diversity and richness, as determined by the Shannon index (**Figure 4A**) and Richness index (**Figure 4B**). Echoing the trends seen in **Figure 3A**, the Rarefaction curve suggested lower Shannon index values (**Figure 4C**) and a reduced count of OTUs (**Figure 4D**) in the orlistat-treated mice. These findings suggest that a 60% fat diet correlates with increased microbial diversity and richness, while orlistat administration can diminish these parameters. The microbial composition of fecal samples from the A, F, and N groups was comparatively analyzed, and bacteria of relatively high abundance are illustrated in **Figure 5**. A notable decrease in *Bacteroidetes* proportion was noted in mice fed on a 60% fat diet, which was further reduced by orlistat treatment. In contrast, the *Helicobacter* proportion declined in the F group, whereas orlistat treatment was linked to a higher abundance of *Helicobacter*. Additionally, the proportion of *Allobaculum* increased in response to a 60% fat diet, with a subsequent increase upon orlistat administration.

**Figure 6:**
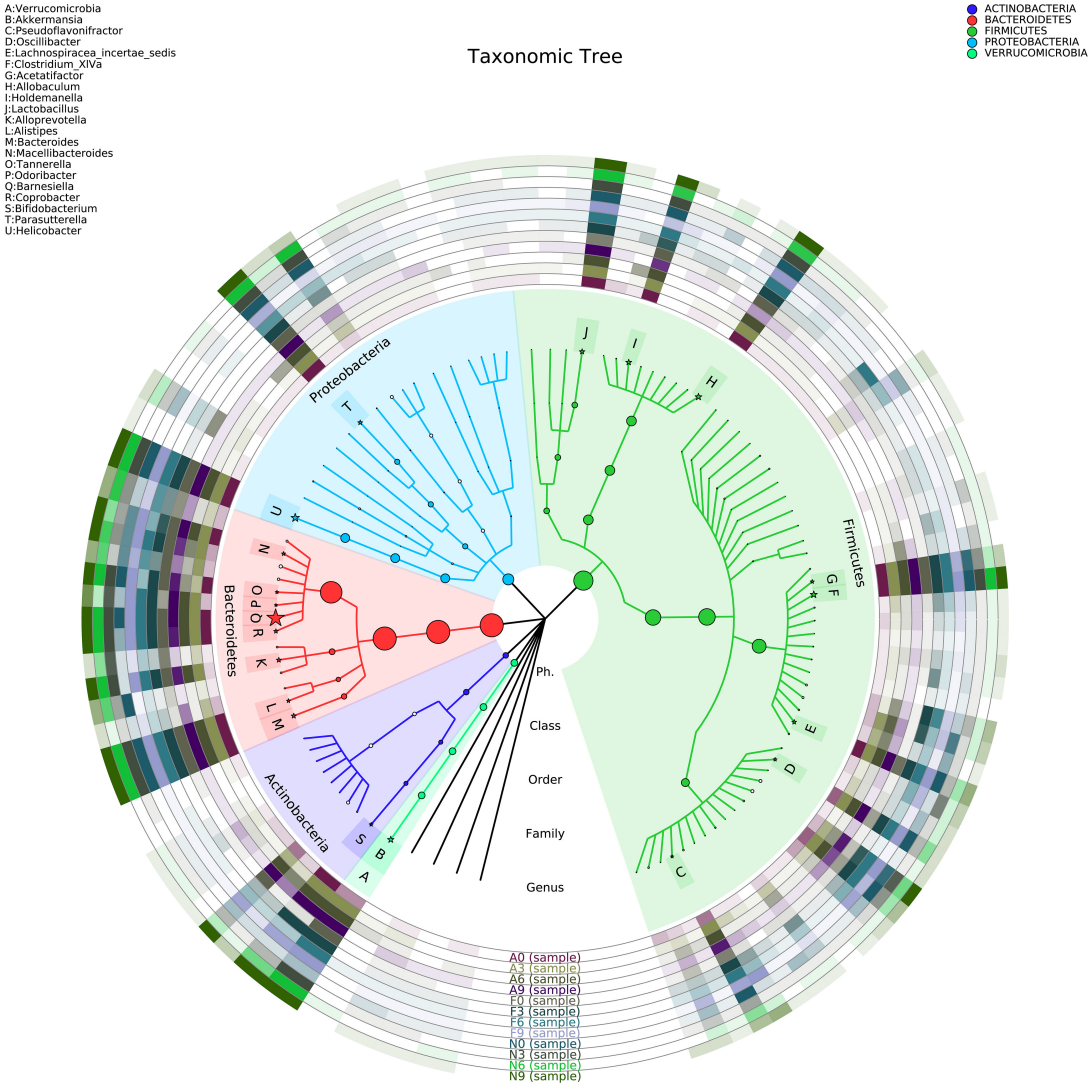
Phylogenetic tree representing the gut microbial composition of fecal samples across three groups. The phylogenetic tree was constructed using MUSCLE (Version 3.8.31) for multiple sequence alignment, and the resulting tree was visualized using R software (Version 3.2).

**Figure 6** illustrates the taxonomic tree of gut microbial composition, revealing shifts in the abundance of *Bacteroidetes*, which bifurcate into two branches. These branches include genera such as *Macellibacteroides*, *Tannerella*, *Odoribacter*, *Barnesiella*, *Coprobacter*, *Alloprevotella*, *Alistipes*, and *Bacteroides*.

**Figure 7:**
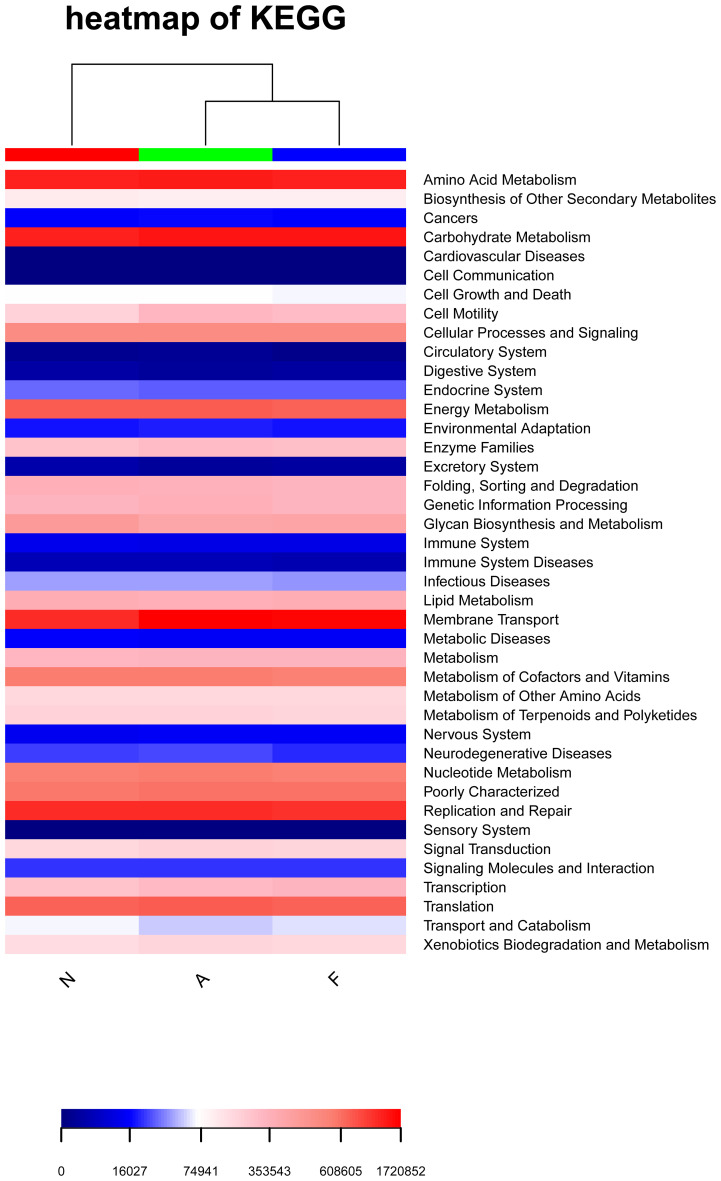
Heatmap illustrating enriched KEGG metabolic pathways from the clustering of the three groups. The heatmap was generated using R software (Version 3.2), and the enrichment analysis was performed using the clusterProfiler package.

Functional variations in the gut microbiota across the groups are depicted in **Figure 7**. Notably, the “cell motility” and “Neurodegenerative Diseases” pathways were substantially enriched in the orlistat-treated mice, whereas the “Glycan Biosynthesis and Metabolism” and “Transport and Catabolism” pathways were less expressed in these mice.

**Figure 8:**
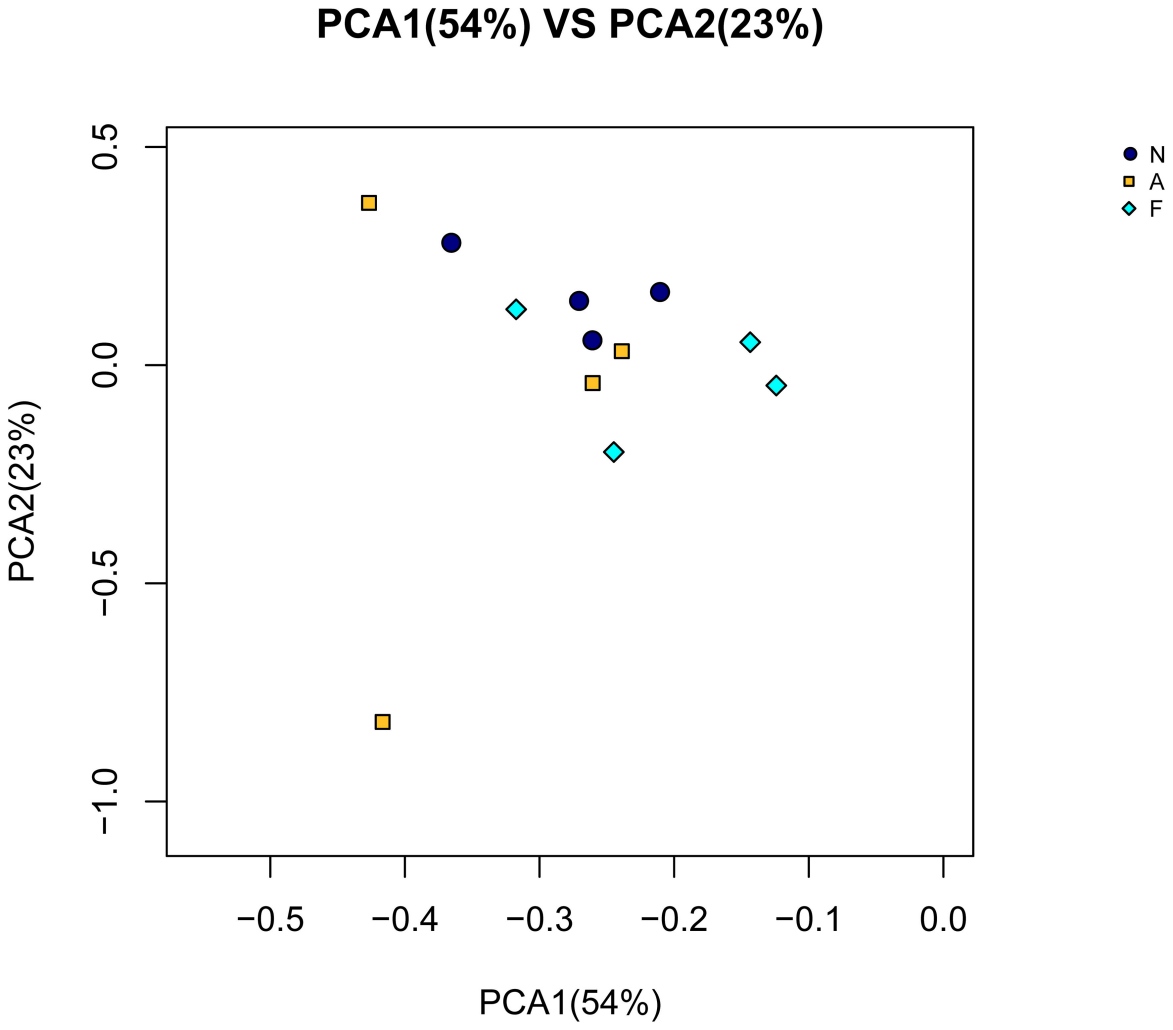
Principal component analysis (PCA) for the similarity of OTUs. PCA was performed using the vegan package in R (Version 3.2) to reduce the dimensionality of the dataset while preserving the most influential features.

**Figure 9:**
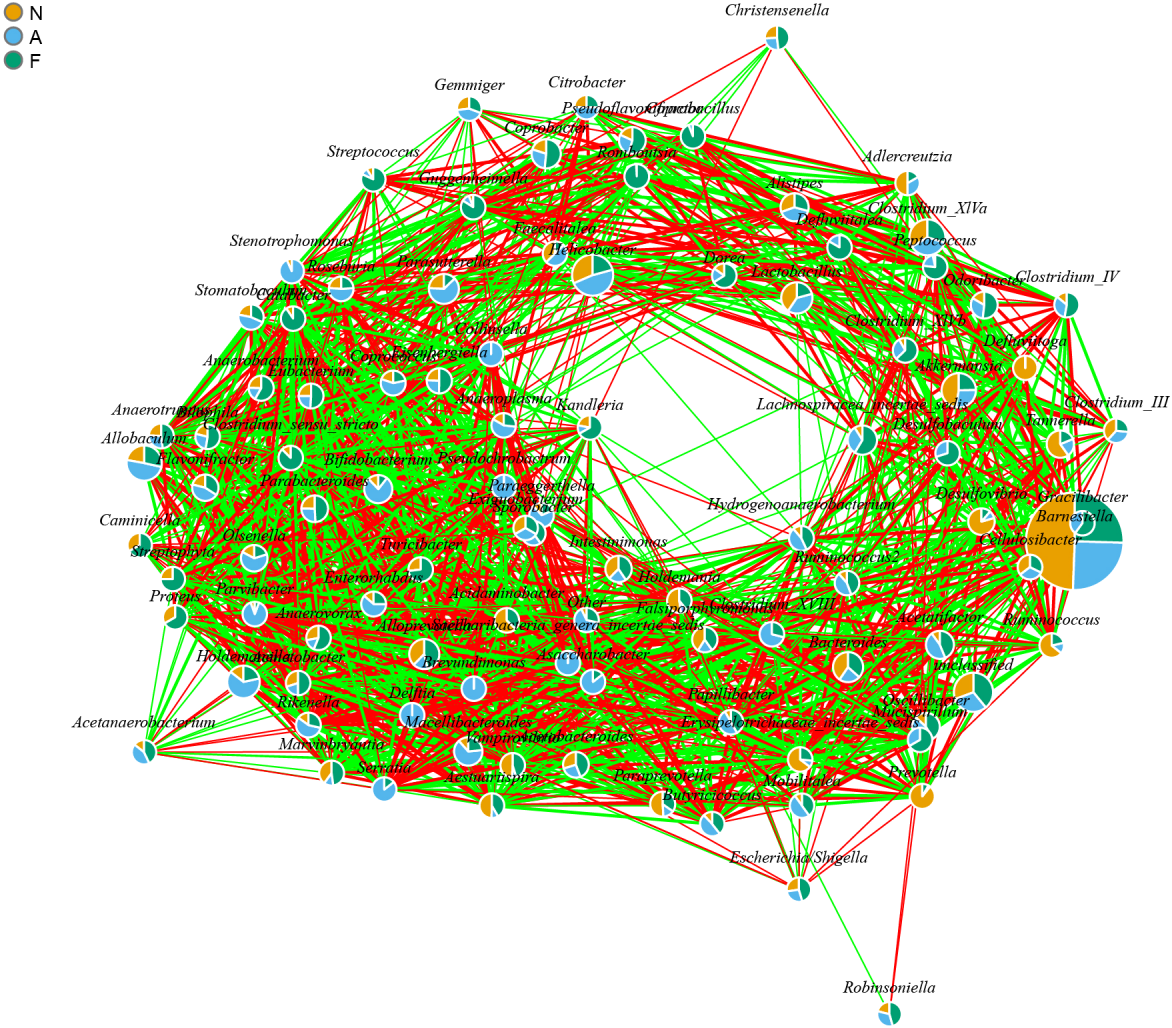
Bacterial co-occurrence network analysis highlighting the relationships associated with a high-fat diet and orlistat treatment. Spearman correlation analysis was performed using non-rarified sequence data, and edges were created between bacterial genera if *P* < 0.05 and |r| > 0.7. The network was visualized using the igraph package in R (Version 3.2).

The PCA outcomes, shown in **Figure 8**, indicate alterations in the OTU similarity in the F group, which appeared to normalize following orlistat treatment. Spearman’s rank correlations were employed to examine the co-occurrence patterns of microbial communities at the phylum level, aiming to discern shifts in bacterial ecosystem structure (**Figure 9**). A relatively intricate network of correlations was observed in the microbiota of orlistat-treated mice, in contrast to a more simplified network in mice fed on a 60% fat diet. Orlistat was also correlated with a reduced density in the bacterial correlation network.

**Figure 10:**
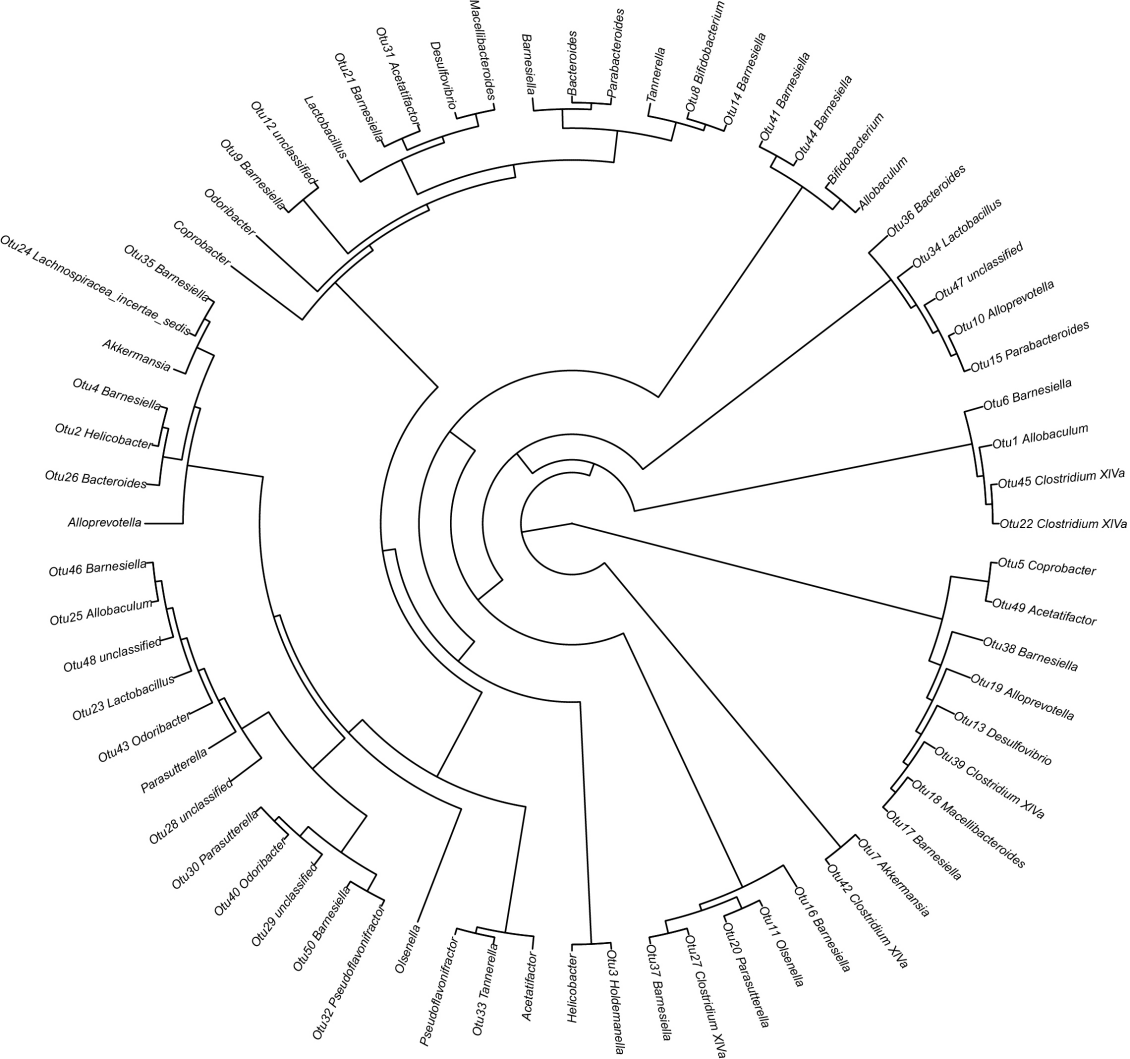
Phylogenetic tree detailing the gut microbial composition of fecal samples. The phylogenetic tree was constructed using MUSCLE (Version 3.8.31) for multiple sequence alignment, and the resulting tree was visualized using R software (Version 3.2).

**Figure 11:**
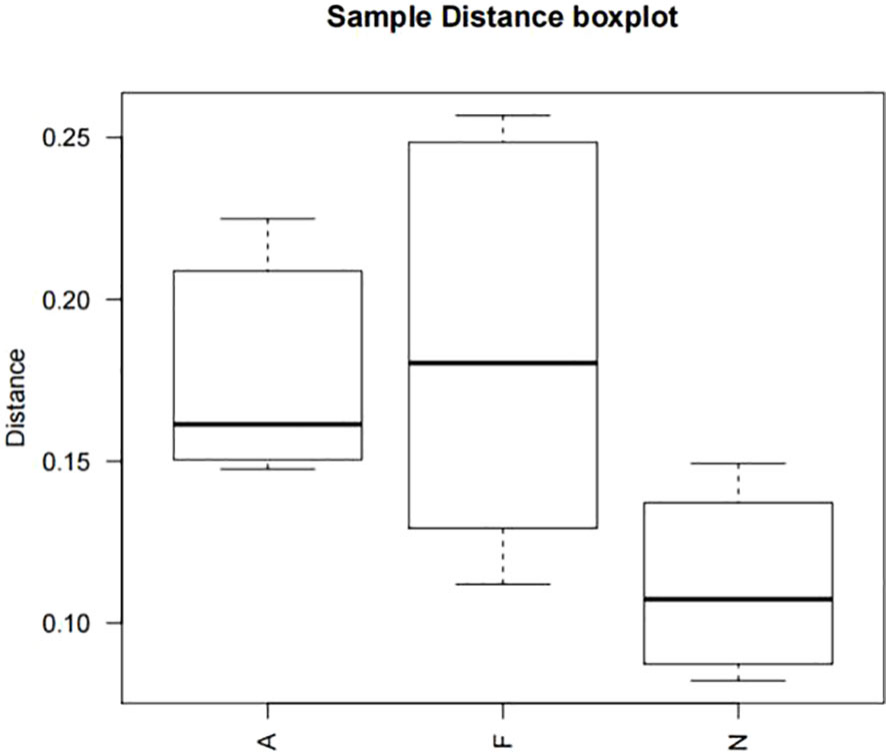
Boxplot of sample distances for multiple comparisons across three groups. One-way ANOVA was applied to compare the distances among the different groups, followed by *post-hoc* pairwise comparisons using the Bonferroni method. All P values are two-tailed, with a significance threshold of 0.05.

**Figure 12:**
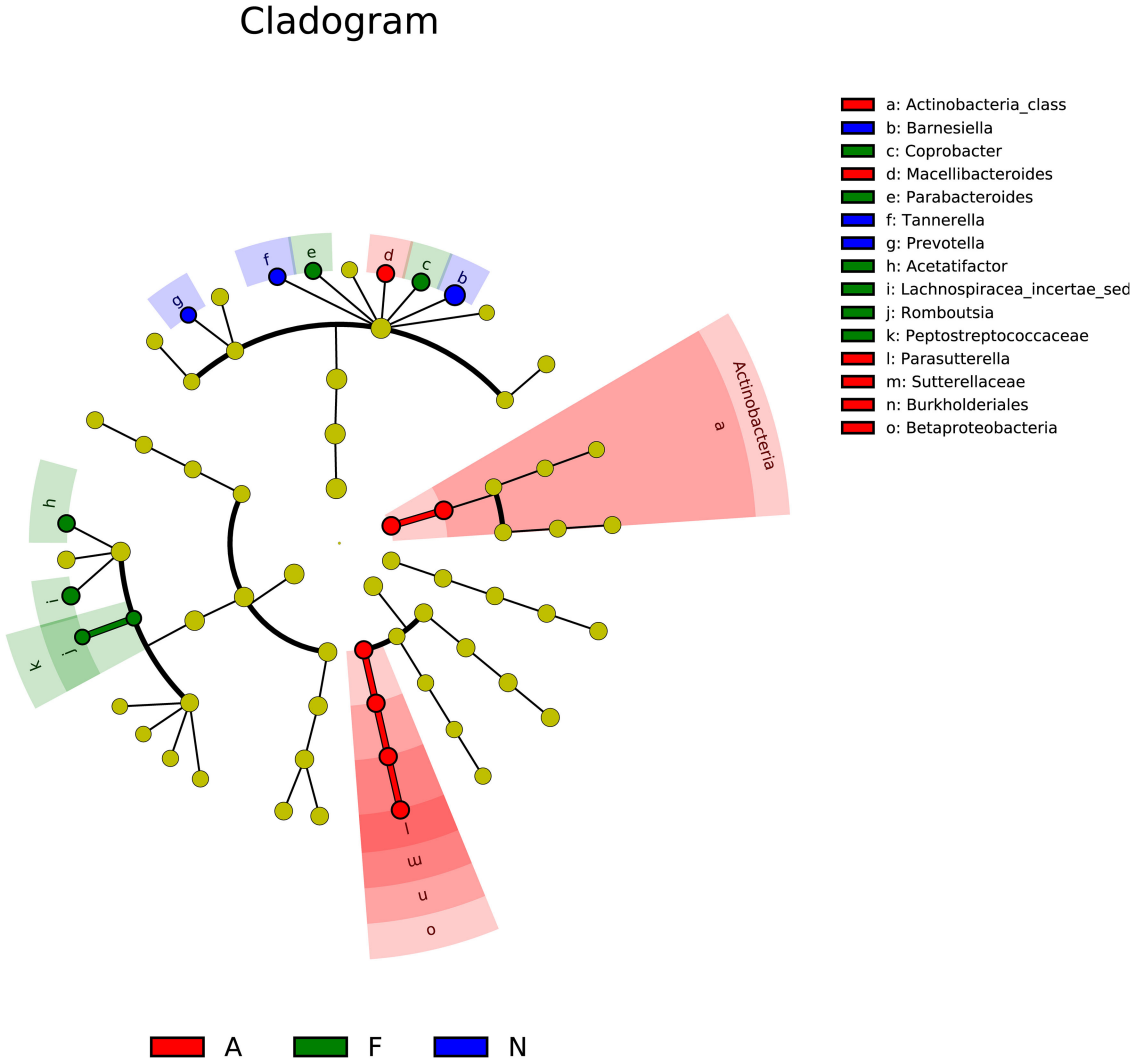
Linear discriminant analysis effect size (LEfSe) for multiple samples among the three groups. The analysis includes two main steps: (1) Kruskal-Wallis rank sum test to detect features with significantly different abundances between groups, and (2) Linear Discriminant Analysis (LDA) to estimate the effect size of each feature. Features with LDA scores greater than 2.0 were considered significant. The LEfSe analysis was performed using the lefse tool in the Galaxy platform.

The phylogenetic tree results are displayed in **Figure 10**. Distances between multiple samples within the three groups were computed, and the variance in these distances was assessed within as well as across groups (**Figure 11**). The findings indicated an expanded sample distance within the model group, while the range of fluctuation in the A group was akin to that of the N group. The LEfSe results highlighted the significance of *Coprobacter*, *Parabacteroides*, *Acetatifactor*, *Lachnospiraceae*, *Romboutsia*, and *Peptostreptococcaceae* in mice on a 60% fat diet. After orlistat treatment, differences were observed in *Actinobacteria*, *Macellibacteroides*, *Parasutterella*, *Sutterellaceae*, *Burkholderiales*, and *Betaproteobacteria* (**Figure 12**).

## Discussion

Obesity is mediated by chronic inflammation, stemming from an imbalance in the body’s energy homeostasis—where energy intake surpasses energy expenditure. In this study, obesity was successfully induced in mice fed on a 60% fat diet. The administration of orlistat, however, significantly curtailed the average weekly weight gain and normalized FPG levels. It was observed that a 60% fat diet resulted in decreased levels of GLP-1 and GIP, whereas orlistat use led to an increase in these hormones, regardless of body weight changes. Notably, an imbalance in gut microbiota is intricately linked to obesity (36). Our research investigated the effects of orlistat on the gut microbiota in obese mice. Key findings include: (1) orlistat significantly reduced the enhanced microbial diversity and richness caused by the high-fat diet; (2) the proportion of *Bacteroidetes* decreased with the high-fat diet and was further reduced by orlistat; (3) *Helicobacter*, which decreased with the high-fat diet, increased with orlistat treatment; (4) *Allobaculum* increased with the high-fat diet and was further augmented by orlistat; (5) orlistat use correlated with enrichment in “cell motility” and “Neurodegenerative Diseases” pathways; (6) orlistat normalized the altered OTU similarity observed in high-fat diet mice; and (7) co-occurrence network analysis showed a more complex but less dense bacterial network in orlistat-treated mice.

Several studies have highlighted the modified role of gut microbiota in mice following a high-fat diet (37–39). Ke et al. aimed to evaluate the influence of orlistat on the gut microbiota of C57BL/6J obese mice fed with a high-fat diet. Their findings suggested that orlistat improved body weight, plasma cholesterol, and glucose tolerance, while also reducing microbial abundance (37). Jin et al. investigated the anti-obesity effects of orlistat and ezetimibe, revealing that these drugs exert distinct anti-obesity effects by modulating the gut microbiota in different ways (38). Deng et al. reported an association between increased water intake, serum triglyceride levels, lower glucose tolerance, and the genus-level abundance of *Roseburia*, *Bacteroides*, *Faecalibacterium*, *Coprobacillus*, and *Akkermansia*, noting differences between the effects of psyllium husk and orlistat (39). Despite these valuable insights, the specific mechanisms by which orlistat affects the gut microbiota and subsequently influences intestinal hormones in obese mice have not been fully elucidated. Our study addresses this gap by demonstrating that orlistat modulates the gut microbiota, leading to beneficial effects on body weight, FPG levels, and the hormones GLP-1 and GIP.

Our study confirmed that orlistat intake correlated with reduced body weight and FPG levels, aligning with findings from published research studies. This correlation might be attributed to enhanced insulin sensitivity following weight loss (40). Additionally, we noted a decrease in intestinal hormone levels in mice fed on a 60% fat diet but observed an increase in GLP-1 and GIP levels with orlistat administration. Several factors could be responsible for these outcomes. First, GLP-1 can suppress appetite via the central nervous system, slowing down gastric emptying and promoting satiety. Furthermore, elevated GLP-1 levels can improve the function of islet cells, contributing to body weight reduction. The association of increased GLP-1 levels with orlistat usage implies a significant role for the GLP-1 pathway in orlistat’s impact on body weight (41). Second, GIP downregulation is linked to diminished insulin secretion. However, orlistat may modulate GIP’s biological activity, influencing FPG outcomes (42).

Regarding the composition of gut microbiota, an increase in microbial diversity and richness was observed in mice fed on a 60% fat diet. This observation is in contrast to findings from a previous study (37). The relationship between microbial diversity, richness, and high-fat diets is complex; our study utilized a specific 60% fat diet (including 2 kg of complete diet, 1.2 kg of lard, 100 g of milk powder, 0.8 kg of maltose, etc.), which varied from the diet employed in the referenced study (37). Consistent with the prior study (37), orlistat administration was linked to a reduction in microbial diversity and richness. Additionally, obese mice treated with orlistat showed an increased presence of *Actinobacteria* and *Proteobacteria*. The administration of orlistat also led to a decrease in the proportion of *Bacteroidetes*, known for their immunomodulatory effects on the host. The dysbiotic microbiota in obesity may enhance energy harvest, possibly through the suppression of angiopoietin-like protein 4, contributing to increased adiposity in the host (8, 43). We identified members of *Bacteroidetes* such as *Macellibacteroides*, *Tannerella*, *Odoribacter*, *Barnesiella*, or *Coprobacter*, as well as *Alloprevotella*, *Alistipes*, or *Bacteroides* in our samples. Additionally, obese mice administered with orlistat showed functional shifts in the gut microbiota, with gene enrichment in pathways related to “cell motility” and “Neurodegenerative Diseases.” These changes may be linked to orlistat’s effect on fatty acid synthase levels, crucial for orlistat’s positive outcomes (44). Moreover, as orlistat inhibits gastric and pancreatic lipases without being absorbed, it is associated with diminished vitamin D absorption, which has a significant role in lipid metabolism and is relevant to obesity (45, 46).

The LEfSe analysis indicated that orlistat usage could alter the composition of various bacterial taxa, including *Actinobacteria*, *Macellibacteroides*, *Parasutterella*, *Sutterellaceae*, *Burkholderiales*, and *Betaproteobacteria*. *Actinobacteria* are known to influence metabolism by modulating intestinal cholesterol absorption, enhancing triglyceride synthesis, and diminishing glycogenesis in the liver (47). The impact of ω3-fatty acids on host physiology might be mediated by *Parasutterella* (48), which is also associated with triggering inflammatory responses in the intestinal mucosa and systemic metabolic irregularities; moreover, *Parasutterella* dysbiosis may lead to low-grade metabolic inflammation (49, 50). Additional research is necessary to confirm the potential role of these and other bacterial genera in obesity.

While our study provides valuable insights into the effects of orlistat on the gut microbiota in obese mice, several limitations should be acknowledged. First, our study was conducted in a murine model, and the results may not be directly translatable to human subjects. Second, the duration of the orlistat treatment was limited, and long-term effects of orlistat on the gut microbiota were not evaluated. Third, while we observed changes in microbial diversity and specific bacterial taxa, the functional implications of these changes were inferred through pathway analysis. Finally, the co-occurrence network analysis, although informative, is based on correlation and does not establish causality. Further experimental validation is needed to confirm the interactions and functional relationships within the bacterial communities.

## Conclusion

This study revealed that orlistat induces beneficial effects on body weight, fasting blood plasma glucose, and the hormones GLP-1 and GIP via modulating gut microbiota. Additionally, the proportions of Actinobacteria and Proteobacteria were higher in obese mice treated with orlistat. Conversely, the proportion of *Bacteroidetes*, including the two branches—*Macellibacteroides*, *Tannerella*, *Odoribacter*, *Barnesiella*, and *Coprobacter*; *Alloprevotella*, *Alistipes*, and *Bacteroides*—was reduced. Future research should investigate the specific mechanisms by which orlistat affects the gut microbiota in obese mice, induced via a high-fat diet.

## Data Availability

The data presented in the study are deposited in the NMDC repository: Submission Number. SUB1740036136174; Biological Project Number, NMDC10019750; Biological Sample Number, NMDC20366075; Multivariate omics data number, NMDCM0001025.
